# BODIES: BOdy shape parameter and 3D meshes of Individuals basEd on SUPR

**DOI:** 10.1038/s41597-026-06777-4

**Published:** 2026-03-24

**Authors:** Alberto Cannavò, Francesco Manigrasso, Federica Moro, Fabrizio Lamberti

**Affiliations:** Department of Control and Computer Engineering, Politecnico di Torino, Turin, Italy

**Keywords:** Computational science, Software

## Abstract

Today, an increasing number of applications in domains such as cultural heritage, healthcare, education, entertainment, and fashion require high-fidelity 3D avatars. However, generating avatars that faithfully reproduce users’ bodies through modeling or acquisition techniques remains challenging and time-consuming, particularly in applications where the accurate quantitative reproduction of body shape and precise anthropometric measurements is required. Thus, attention is shifting towards machine learning-based approaches, in particular those able to fit a parametric model representing the avatar to the intended body shape. Among these models, the Sparse Unified Part-Based Human Representation (SUPR) has been proven to offer superior performance compared to other representations. However, its adoption is primarily hindered by the lack of datasets built upon it. This paper addresses this gap by proposing *BOdy shape parameter and 3D meshes of Individuals basEd on SUPR (BODIES)*, a dataset containing 84,000 synthetic-generated subjects described using the SUPR model with different numbers of parameters. The paper also presents the results of three experimental studies aimed at assessing the improvements brought by the SUPR model over the state-of-the-art when used to feed an existing framework for generating 3D avatar meshes.

## Background & Summary

Based on recent reviews^1^, the availability of high-fidelity 3D meshes of humans has become a pivotal element in multiple tasks like human body modeling^2^ and reconstruction^3^, mesh recovery^4^, body and pose estimation^5^, animating clothed 3D avatars^6^, etc. Today, the majority of these tasks heavily rely on machine learning (ML) techniques to generate, manipulate, or analyze 3D human representations.

In this respect, among the numerous parametric models recently proposed in the literature like, e.g., Neural Body^7^ and GHUM & GHUM-L(ite)^8^, the one that has become the *de facto* standard for representing 3D human bodies^9^ is the Skinned Multi-Person and Linear (SMPL) model^10^. The SMPL model is based on a template mesh (composed of 6,890 vertices) that is deformed by means of a skeleton (featuring 23 joints) and of blendshapes aimed at changing the 3D mesh topology (e.g., to control subject’s height, weight, waist size, shoulder width) or correcting it when the avatar assumes a new pose.

Works such as^2,11,12^ showed that a high reconstruction accuracy can be obtained by leveraging SMPL or the SMPL eXpressive (SMPL-X) extension. However, despite their widespread use, those above are not the most recent models for human mesh representation available to date.

A relatively new parametric model, named Sparse Unified Part-Based Human Representation (SUPR)^13^, was recently proposed. SUPR is able to capture better than SMPL (and its extension) the full range of motions and deformations, especially for the head and hands, thanks to the 4D full-body scans used for the training. Moreover, SUPR adds the representation of the feet, which is largely ignored by previous models, thus enabling the description of new movements (e.g., deformations due to ground contact) of this body part.

The model was trained by using 1.2 million scans of the above parts. To make it better generalize, training data contain extreme body shapes (such as patients suffering from anorexia and bodybuilders), 14,000 records from the Civilian American and European Surface Anthropometry Resource (CAESAR) and SizeUSA datasets, and 7,000 feet records from the Anthropometric Survey of US Army Personnel (ANSUR II) dataset^14^.

**Fig. 1:**
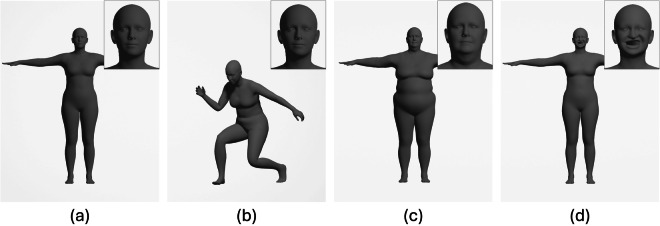
Subject represented with the SUPR model: (**a**) template mesh and sample deformations achieved using (**b**) pose, (**c**) shape, and (**d**) expression parameters.

SUPR consists of a 3D template mesh, shown in Fig. 1a, which leverages linear blend skinning (LBS) and supports blendshape-based deformation. The number of vertices in the mesh is the same of SMPL-X (10,475), while the number of joints in the skeleton is only slightly larger (75 compared to 54, especially used to control feet, ankles, and toes). Nevertheless, thanks to the adopted separation of body parts as well as to the quality and variety of data used for creating it, SUPR is capable of achieving significantly higher representation performance compared to previous models.

Indeed, based on the above considerations, it is reasonable to expect that the use of the SUPR model would bring numerous advantages, especially in terms of reconstruction accuracy over previously adopted models. Improvements in terms of reconstruction accuracy are particularly important in applications where faithful extraction and reproduction of body shape and anthropometric measurements is critical, including the creation of personalized avatars for entertainment, realistic virtual try-on applications in fashion, patient-specific models in healthcare, and accurate guides or characters in virtual heritage experiences (more details are provided in Section “Use Cases”).

Nevertheless, the use of this model is still very limited. The results of a literature review conducted by analyzing the main academic databases including Scopus, Web of Science, and Google Scholar show that, to date, there are 33 papers citing SUPR compared to 5461 papers citing SMPL. Among the papers citing SUPR, only four explicitly consider its use. More specifically, two of them (i.e.,^15^ and^16^) employ it for 3D human model generation and animation, whereas the others (i.e.,^17^ and^18^) reference SUPR only theoretically as a potential solution to the limitations of their proposals, without providing a practical implementation. Regarding the remaining works, 20 of them adopted SMPL or its variants, further reinforcing the prevalence of these models.

Reasons behind the limited use of SUPR may be related to the lack of available datasets that can be used as benchmarks to validate and test ML-based solutions aimed at generating high-fidelity 3D human meshes, rather than to train novel models^19^. This limitation is particularly crucial as it has been demonstrated, e.g., in studies like^20^, that the strategic selection and use of high-quality datasets incorporating critical attributes (e.g., shape features of the humans) can yield a significant boost in the performance of ML-based solutions.

Datasets such as Chest, wAist and peLVIS circumference from 3D human Body meshes for Deep Learning (CALVIS)^21^, Synthetic hUmans foR REAL tasks (SURREAL)^22^, AGORA: Avatars in Geography Optimized for Regression Analysis (AGORA)^23^, Archive of Motion Capture As Surface Shapes (AMASS)^24^, CAPE: Clothed Auto Person Encoding^25^, HuGe100K^26^, and MVHumanNet^27^ are examples of widely adopted datasets that still rely on the SMPL model (or its extension). A comparison of these datasets is reported in Table 1. Moreover, to the best of the authors’ knowledge, tools able to convert existing datasets based on representations like SMPL/SMPL-X to SUPR without introducing biases or errors do not exist yet.

**Table 1:** Summary of human body datasets.

Dataset	Year	Model	Subj. #	Dataset Description
SURREAL	2017	SMPL	145	6.5 million synthetically-generated RGB images together with 2D/3D poses, surface normals, optical flow, depth images, and body-part segmentation maps for rendered people
CAPE	2017	New model to extend SMPL	11	80K frames of subjects performing a variety of pose sequences in different types of clothing captured using a 4D scanner
AMASS	2019	SMPL	344	40 hours of motion capture data. Each frame includes the SMPL parameters
CALVIS	2019	SMPL	3803	3D human body meshes, annotations (chest, waist, and pelvis circumference), synthetic images
AGORA	2021	New model to extend SMPL-X	4240	17K images created by rendering 5-15 people per image in varied clothing. Each image includes reference 3D meshes (poses and body shapes) obtained by fitting the SMPL-X body model (with face and hands) to the 3D scans as well as person-person occlusion, environmental occlusion, camera frame occlusion map
MVHumanNet	2024	SMPL/ SMPL-X	4,500	9,000 daily outfits, 60,000 motion sequences and 645 million frames with extensive annotations, including human masks, camera parameters, 2D and 3D keypoints, SMPL/SMPL-X parameters, and corresponding textual descriptions
HuGe100K	2025	SMPL-X	100K	2.4M high-resolution multi-view images with SMPL-X parameters of the 100K subjects
Ours	2025	SUPR	84K	3D mesh, annotations (weight, height), SUPR shape parameters, front-/side rendered image for each subject

Another limitation that could hinder the adoption of SUPR is the lack of comparative studies aimed at demonstrating its effective superiority over existing alternatives. In fact, besides the evaluation reported in the paper originally presenting the SUPR model^13^, there are no further examples of comprehensive experiments or benchmarks comparing SUPR to other leading models across different tasks, datasets, or performance metrics. This absence of additional validation makes it difficult to assess its true potential and advantages in practical applications.

The aim of the present work is to fill these gaps by introducing a synthetic data generation technique to build a dataset consisting of subjects described using the SUPR model.

The dataset is named *BOdy shape parameter and 3D meshes of Individuals basEd on SUPR (BODIES)*, and contains 84,000 subjects described with different numbers of parameters. The dataset is available for download at^28^.

In order to evaluate the advantages of the SUPR model, we also conducted three experimental studies, focusing on the task of ML-based human mesh reconstruction. For all the studies, one of the existing frameworks for the generation of human meshes (precisely, the one presented in^2^) was used. The first study was aimed to directly compare the performance of SUPR over SMPL, by evaluating the reconstruction error obtained with the said framework when the proposed dataset is used as the training set. The second study investigated how the reconstruction error varies based on the number of model parameters used to represent the human body. Finally, the ability to reconstruct the body of real subjects has been analyzed by comparing the results achieved using the proposed dataset against those obtained using existing ones from both objective and subjective perspectives.

## Methods

According to^13^, the SUPR model can be formally expressed through the function *M*(⋅) defined as

1 $$M(\overrightarrow{\theta },\overrightarrow{\beta },\overrightarrow{\psi })=W({T}_{P}(\overrightarrow{\theta },\overrightarrow{\beta },\overrightarrow{\psi }),J(\overrightarrow{\beta }),\overrightarrow{\theta };\omega )$$

where:

- $\overrightarrow{\theta }\in {{\mathbb{R}}}^{75\times 3}$ are the *pose parameters*, that correspond to the 3D rotations of the 75 joints. An example of mesh deformation obtained by using the pose parameters is depicted in Fig. 1b. These parameters also influence pose-corrective blendshapes that are included in the model to limit LBS artifacts, such as the “candy-wrapper” effect^29^.
- $\overrightarrow{\beta }\in {{\mathbb{R}}}^{300}$ are the *shape parameters*, i.e. the 300 descriptors of the body shape that characterize the aspect of the subjects. These parameters control the shape blendshapes used to deform the mesh. Generally, only a limited set of these descriptors is used, since the first 10 parameters already include most of the variations in the shape (e.g., height, weight, waist size, shoulder width). The use of a larger number of parameters, though, provides more granular control over specific body characteristics. An example showing the influence of shape parameters is given in Fig. 1c.
- $\overrightarrow{\psi }\in {{\mathbb{R}}}^{100}$ are the *expression parameters* that control the deformation of the face through corresponding facial blendshapes. Deforming the mesh with expression parameters results in facial expressions such as the one shown in Fig. 1d.
- *T*_*p*_(⋅) is a function that deforms the template mesh depending on the values of the $\overrightarrow{\theta }$, $\overrightarrow{\beta }$, and $\overrightarrow{\psi }$ parameters.

In the present work, the focus is on estimating the $\overrightarrow{\beta }$ parameters of the SUPR model, which contain the information needed to describe the body shape of the subjects of interest. Since posing the subjects, e.g., for animating them, is out of the scope of the work, the parameters regarding poses ($\overrightarrow{\theta }$) and facial expressions ($\overrightarrow{\phi }$) are not considered, assuming that subjects are generated in a static and neutral pose, i.e, T-pose.

**Fig. 2:**
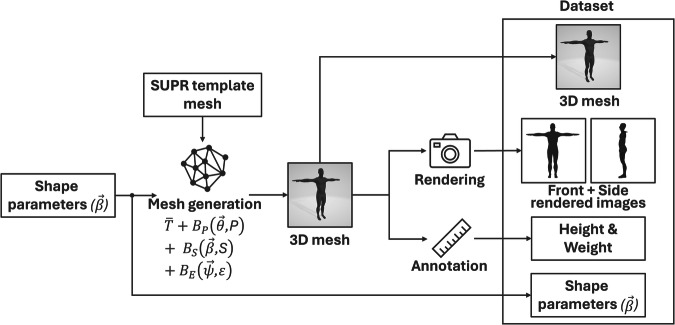
Pipeline for generating the BODIES dataset. The shape parameters $\overrightarrow{\beta }$, randomly generated by following a Gaussian distribution, are used to create a 3D mesh according to the SUPR formulation. Renderings of the 3D mesh are obtained by setting a front and side point of view. Finally, annotations are collected by measuring the 3D mesh.

The pipeline for creating the BODIES dataset (summarized in Fig. 2) includes three stages: i) generating the 3D meshes for all the subjects, ii) rendering, and iii) annotating them.

During the first stage, 3D meshes are generated. Subjects are described in T-pose, hence the pose can be considered as fixed in the parametric formulation. Based on these considerations, constant values are assigned to both the pose ($\overrightarrow{\psi }$) and expression ($\overrightarrow{\theta }$) parameters.

The parameters controlling the body shape are varied to generate 6,000 male and 6,000 female subjects, each described using 10, 16, 32, 64, 128, 256, and 300 parameters. In the remaining of the paper, the label BODIES-*X* is used to indicate the part of the dataset that has been generated using *X* parameters (e.g., BODIES-10 contains subjects described using 10 parameters).

A Python script was created to automatically set the shape parameter values. Different 3D meshes (with distinct body shapes) are generated by deforming the template mesh according to the value of the considered set of parameters. More specifically, each subject was generated by sampling the shape parameters vector $\overrightarrow{\beta }$ = [*β*_1_, *β*_2_, ⋯  , *β*_*N*_], where **N** ∈ {10, 16, 32, 64, 128, 256, 300} depending on the chosen set of parameters used to describe the subjects. Each value *β*_*i*_ was drawn from a Gaussian distribution with mean 0.0 and standard deviation 2.0, and then clipped to the range [−4,4] to prevent the generation of unrealistic body shapes.

This choice was made considering that natural phenomena such as the distribution of the humans’ height and weight within the world population can be described in this way^30^. This simplification may, however, underrepresent certain body types or morphological variations. To account for potential bias introduced by this assumption, future work will involve comparing the generated distributions of key anthropometric measures (e.g., height, weight, limb proportions) with those derived from empirical datasets such as CAESAR or SizeUSA, which include subjects from American and European populations. In this way, potential biases can be quantified and mitigated by adjusting the sampling process, thereby improving representativeness for specific populations.

In order to select which parameters have to be modified it is worth recalling that, in the SUPR parametric model, the first shape parameters already encode the most significant variations in human body shape, while higher-order parameters provide increasingly fine-grained control. For this reason, the generation process begins by varying the first 10 parameters (thus obtaining BODIES-10) and then gradually includes additional ones to reach the desired number of parameters (e.g., BODIES-16). This approach ensures shape diversity (since all generated datasets share the initial core parameters) while maintaining control over the level of detail introduced by granularly considering higher-order parameters. The devised script is also responsible for checking whether duplicate subjects have been generated. This occurs when the newly generated set of values is identical to one previously created. In such cases, a new subject is generated with different parameters to replace the duplicate. The meshes are stored as *.obj* files.

**Fig. 3:**
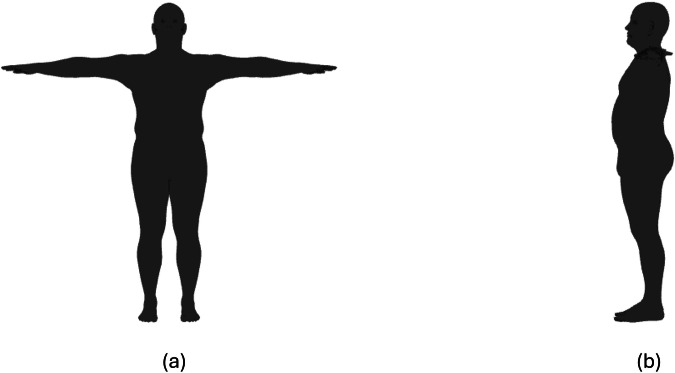
Rendering of a male subject in the BODIES-10 dataset: (**a**) front and (**b**) side view.

In the second stage, rendered images from the front and the side view are produced for each of the generated subjects by means of another Python script. More specifically, the script simulates a 3D scene (containing a virtual camera and a subject) and renders it by leveraging the PyRender, Trimesh, and imageio libraries. To generate the images, the script positions the virtual camera in the origin of the reference system, whereas all the subjects are placed in the same position in front of it. A perspective camera configuration is used with custom intrinsic parameters (focal length X: 1350px, focal length Y: 1350px, optical center X: 250px, optical center Y: 250px, z-near clipping plane: 0.1, z-far clipping plane: 0.1). All the subjects have been rendered without textures in the generated T-pose. The scene does not contain environmental elements or backgrounds. Figure 3 shows an example of a rendered image produced by the script. The images are stored as *.png* files.

In the last stage, a third Python script is used to automatically create annotations for all the subjects. Besides recording the values of the used set of parameters (into a.json file), the height and weight of all the subjects are computed by means of the 3D Body Measurements library (https://tinyurl.com/2akj9srw). More specifically, the functions available in the library compute the height (expressed in meters) of the subjects by extracting a cross-section of the 3D mesh and calculating the distance between the extreme points. The library calculates the weight (expressed in Kilograms) using the formula proposed in^31^:

2 $$Volume(l)=1.015\times Weight(kg)-4.937$$

where *Volume*(*l*) represents the volume of the mesh, computed by leveraging the Trimesh (https://github.com/mikedh/trimesh) library.

**Fig. 4:**
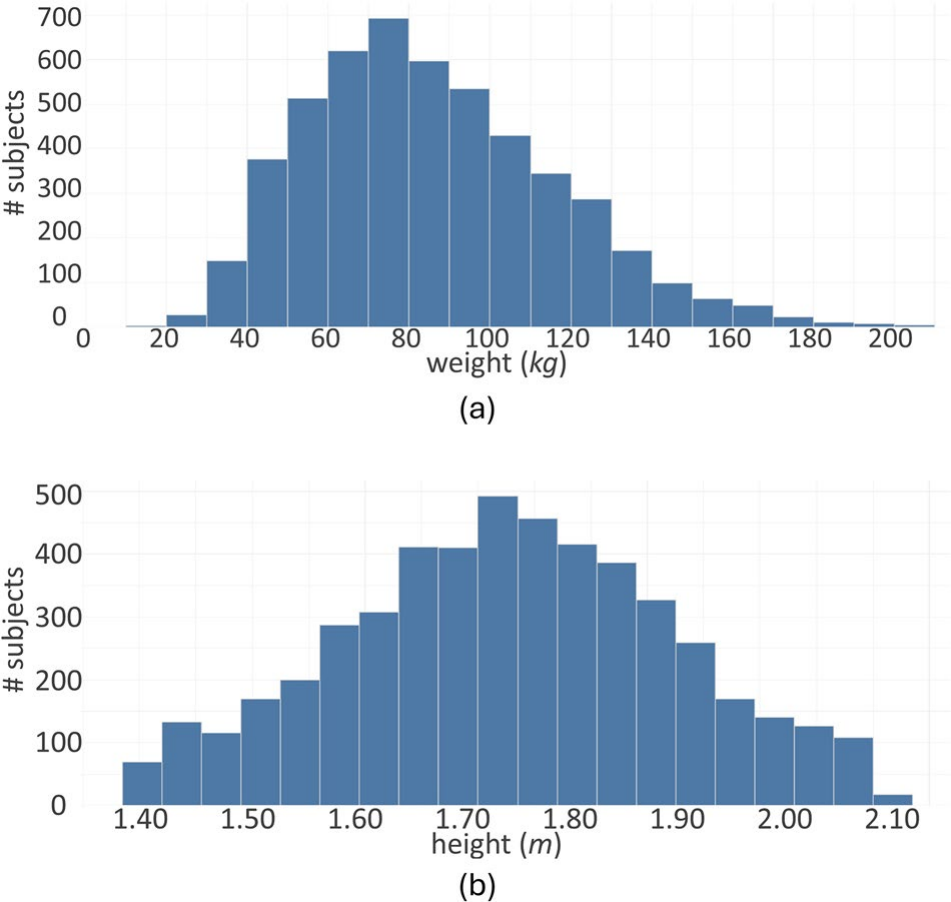
Characteristics of the BODIES-10 dataset: histograms showing the distribution of (**a**) height and (**b**) weight variables (male subjects only).

**Fig. 5:**
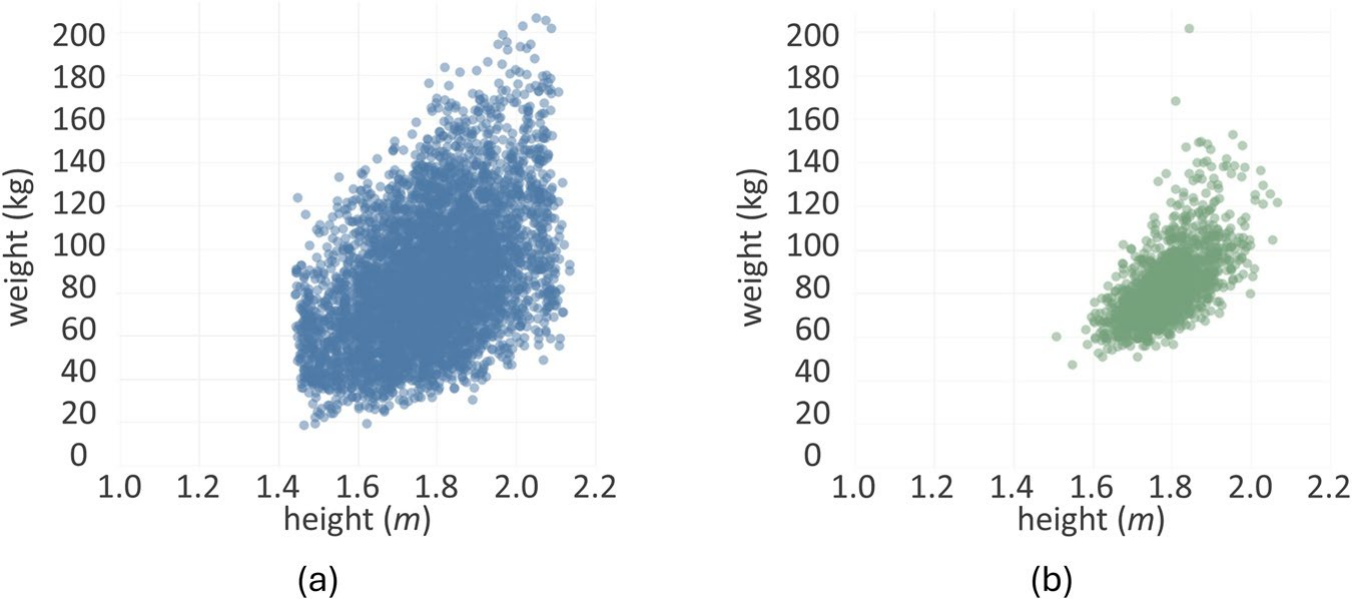
Scatterplots representing the distribution of values for the height and weight variables: (**a**) BODIES-10 and (**b**) CALVIS (male subjects only).

In summary, the BODIES dataset includes two rendered images (front and side views), the 3D mesh, and annotations (values of shape parameters, weight, and height) of 12,000 subjects described with different sets of parameters (84,000 subjects in total). The distribution of weight and height for the male subjects included in the BODIES-10 dataset are reported in Fig. 4a, 4b, respectively. It is possible to observe that the Gaussian distribution of the considered variables has been preserved in the generative process. Figure 5 shows scatterplots illustrating how weight and height variables distribute over the population in the BODIES-10 and CALVIS^21^ datasets (used in the experiments of this work and in^2^). It is worth observing that BODIES-10 exhibits a broader and denser distribution, covering a wider range of body shapes and sizes, whereas CALVIS shows a more compact cluster, indicating a narrower variability among subjects.

**Fig. 6:**
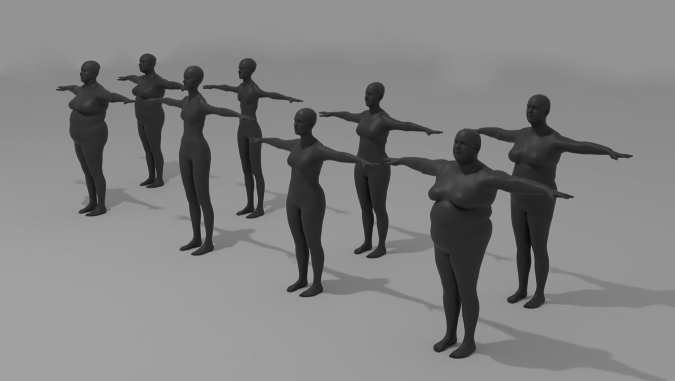
Sample female subjects from the BODIES dataset.

Some examples of female subjects included in the BODIES-10 dataset are provided in Fig. 6.

## Data Records

The dataset introduced in this work^28^ provides data of human subjects described using the SUPR parametric model. For each subject, the dataset offers a parametric representation of the body (i.e., the shape parameters $\overrightarrow{\beta }$), a 3D mesh, front and side rendered images of the subject, and annotations related to the weight and height.

**Fig. 7:**
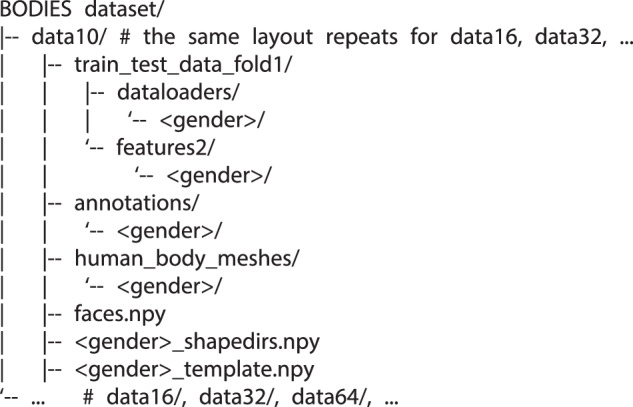
Directory tree of the BODIES dataset.

To guarantee reproducibility, every resource required to replicate the experiments described in the remaining of this work is delivered in a rigidly organised directory tree whose key elements are illustrated in Fig. 7.

Each dataXX folder is a portion of the corpus whose numeric suffix (10, 16, 32, etc.) indicates the number of shape parameters used to represent the subject. The internal hierarchy remains identical across all portions and gender subdivisions. Each portion includes the following folders:

- train_test_data_fold1 contains a folder named dataloader that holds the data referred to the 6,000 subjects already subdivided into training (4,000), validation (1,000), and test (1,000) sets. More specifically, the front and side rendered images are coded in the <set>_512_images.npy. The shape parameters ($\overrightarrow{\beta }$) and body measurements are reported in <set> _betas.npy and <set>_h_w_measures_<gender>_density.npy, respectively. Finally, the file <set>_vertices.npy describes the geometry of the 3D meshes. In addition to the dataloaders, the features2 directory hosts pre-computed higher-level features obtained by using the autoencoder in^2^. These features can be used to downstream experiments that can begin without the need to recompute them.
- annotations contains a *.json* file for each subject in the test set that reports the XX SUPR shape parameters (i.e., $\overrightarrow{\beta }$). These files can be used to speed up the evaluation of the predicted parameters since having direct access to these values removes the need to extract and reconstruct them from the test_betas.npy file.
- human_body_meshes contains the 3D meshes of the subjects in the test set stored as *.obj* files. Meshes are compliant with the SUPR topology and are named using the nomenclature subject_mesh_<ID>.obj.
- <gender>_shapedirs.npy, faces.npy and <gender>_template.npy contain information about the SUPR template and can be used to reconstruct the final 3D mesh as they represent the base values to which the predicted values can be added.

## Technical Validation

To validate the generated dataset, its performance was compared to relevant state-of-the-art datasets when applied to human mesh reconstruction tasks. To this aim, the framework reported in^2^ was considered as a reference to generate the 3D meshes required in the envisaged experimental studies, given its performance in terms of per-vertex mean error. The original architecture of such framework is composed of three main modules:

- An *image segmentation* module based on U-Net^32^ that, starting from a front and a side view image (512  × 512 pixels) of a subject assuming the T-pose, extracts its silhouette.
- An *auto-encoder* module that extracts descriptive image features. Features embedded in the silhouette are extracted and represented more efficiently, reducing their dimensionality.
- A *kernel ridge regressor* module, that receives the features produced by the auto-encoder together with height and weight values, and predicts the first 10 shape parameters of the SMPL model. Shape parameters are used to generate the 3D mesh of the subject. The module also estimates the measurements of the chest, waist, and hip circumferences, which can be used for clothing fit.

**Fig. 8:**
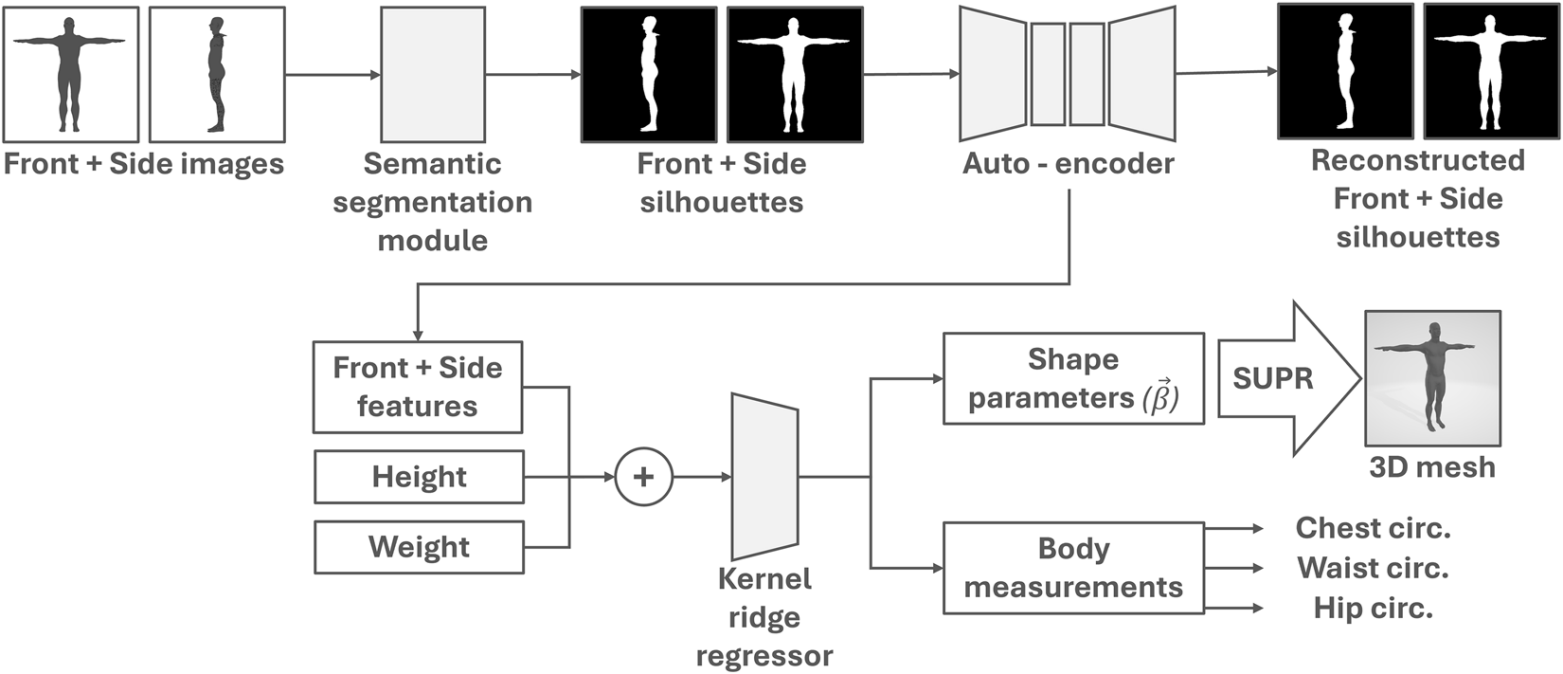
Updated architecture of the framework proposed in^2^ for automatically generating 3D avatar meshes from two input images.

Besides updating the data structure and optimizing the hyperparameters (i.e., learning rate, batch size, number of epochs, and dropout), to make the framework in^2^ support the SUPR model and optimize performance, a number of changes were made (an overview of the updated architecture is shown in Fig. 8).

The first change concerned the Batch Normalization layer in the auto-encoder module. This layer was used to reduce the Internal Covariate Shift. However, works, such as^33^ warned about the limits of Batch Normalization and its effects, and possible alternatives started to be proposed^34,35^. Moreover, in^36^, it was shown that the use of Batch Normalization may introduce artifacts in the generated output. Considering the above aspects, it was chosen to get rid of normalization during the encoding and decoding phases.

**Fig. 9:**
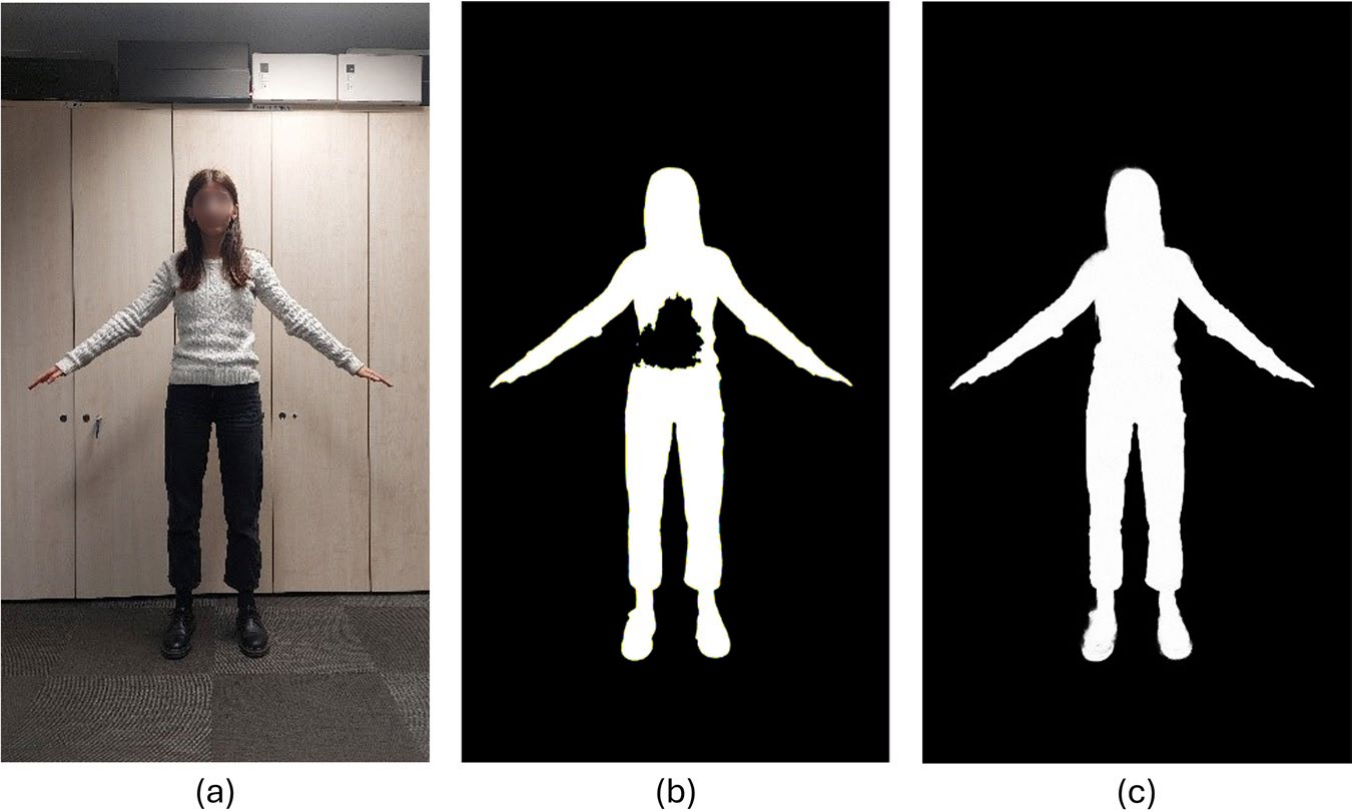
Comparing segmentation methods: (**a**) sample image, (**b**) results with the method used in the reference framework, and (**c**) results with SGHM (used in the optimized architecture).

Another change made to the reference framework regards the image segmentation module. The method used in^2^ was not always able to properly extract the silhouette from the images, as it can be seen by comparing, e.g., Fig. 9a, 9b. For this reason, alternative methods capable to guarantee improved performance were investigated. The Semantic Guided Human Matting (SGHM) framework^37^ was ultimately chosen. The results of image segmentation with SGHM are illustrated in Fig. 9c.

In the experiments, the following parameters and configurations were used: the batch size was set to 32 and the auto-encoder module was trained over 50 epochs. The Adam optimizer was used for training with a learning rate of 0.0001, and image reconstruction performance was evaluated based on average accuracy. The kernel ridge regressor module was trained using a polynomial kernel function with a degree of 3, alpha equal to 1, and optimized with *L*_2_ loss. Experiments were performed on the HPC@POLITO cluster at Politecnico di Torino, equipped with V100 NVIDIA GPUs.

### Experimental Studies

In order to analyze the behavior of the BODIES dataset when used as training and/or test set for the framework in^2^ and compare its performance when the same framework is trained and/or tested with other datasets, the following three studies were devised.

#### Study 1 – Comparing SMPL and SUPR as Training Sets

The goal of this study was to compare the performance of the framework proposed in^2^ when trained with the SMPL and SUPR models and tested with datasets containing human meshes represented with the same or the other model. For what it concerns the SMPL model, the CALVIS dataset^21^ was chosen as done in^2^. For the SUPR model, the BODIES-10 dataset was used, in order to make the comparisons fair, since, in this way, both datasets contain subjects represented with 10 parameters.

The two configurations below were analyzed:

- CONF-1: The optimized version of the framework in^2^ trained with the CALVIS dataset.
- CONF-2: The optimized version of the framework in^2^ adapted to operate with the SUPR model and trained with BODIES-10.

Samples contained in the two datasets (i.e., CALVIS and BODIES) were split into 80% for training and 20% for testing, as done in^2^. This choice was made to make the results obtained in the present work comparable with those reported in the reference work.

**Fig. 10:**
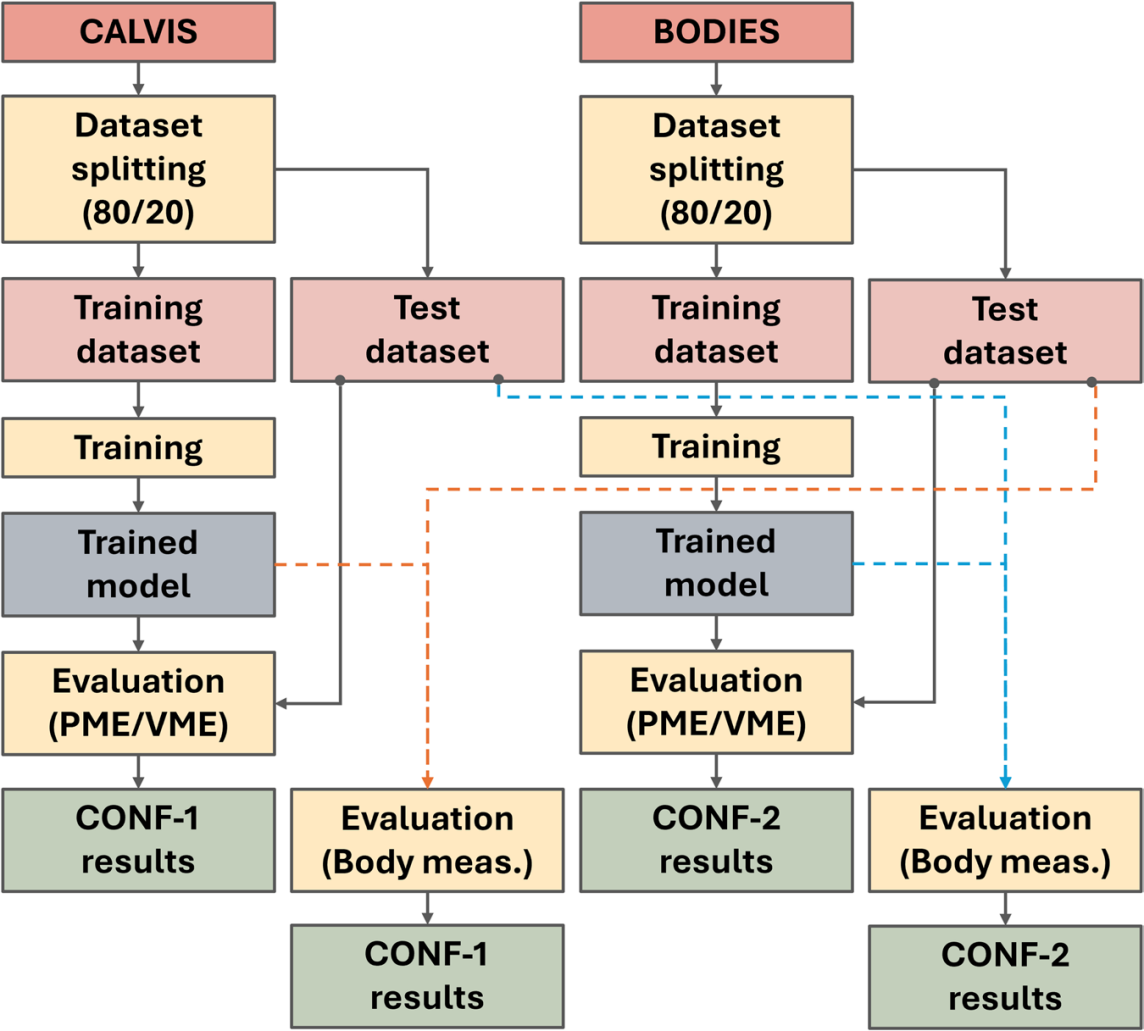
Schematic representation of the training and testing workflow adopted in Study 1.

Figure 10 shows an overview diagram illustrating how the datasets were used for training and testing in this first study.

#### Study 2 – Analyzing the Effect of Varying the Number of Parameters

This study aimed to analyze the impact that a larger set of parameters could have on the final reconstruction of the human meshes. In fact, it is worth observing that an increase in the number of parameters should decrease the reconstruction error (as previously reported in^13^), due to the fact that more parameters should allow the framework to model finer details of the human body meshes. However, the increased number of parameters to be estimated also translates into an increased complexity, which could lead to worse performance.

Differently than in the previous study, where a single 80/20 partition is used, in this study, the entire architecture underwent training using K-fold cross-validation to better understand the generalization capabilities of the framework on test data, as well as to achieve a more precise assessment of model performance. In the experiments, 1,000 samples were randomly chosen to serve as test set. The remaining samples were then used to perform the training in a K-fold cross-validation fashion with K  = 5. The architecture that performed the best during this process was then used for the inference phase.

Also for this study several configurations were defined and tested, in the following indicated with CONF-*X* (with *X* indicating the dataset used as training set). For instance, configuration CONF-BODIES-10 means that performance of the framework was evaluated by using the BODIES-10 as a training set and subjects that could belong to BODIES-16,-32,-64,-128,-256,-300 for the test set. For this study and the following one, the optimized version of the framework was used; hence, no additional configurations were added to consider different versions of the framework.

**Fig. 11:**
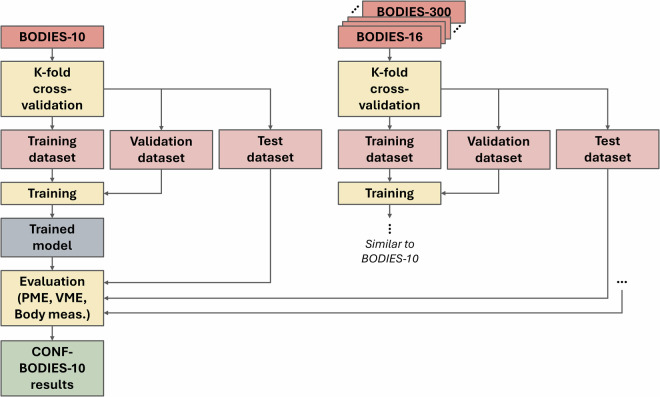
Schematic representation of the training and testing workflow adopted in Study 2.

Figure 11 provides the schematic representation of the dataset usage during the training and testing phases of this second study.

#### Study 3 – Evaluating the Training with SMPL and SUPR for Reconstructing Real Subjects

The goal of the last study was to check whether there are differences due to training performed with the CALVIS or one of the BODIES datasets when the framework is used to reconstruct real subjects. In this study, the impact of the datasets used for training on the final reconstruction of human meshes was evaluated both quantitatively and qualitatively, using the objective and subjective metrics discussed in the following section.

The dataset used as a test set included 34 subjects (17 males and 17 females) that were extracted from the Scan DB dataset^33^. This dataset contains the meshes of real subjects obtained through 3D scanning. The subjects for the study were selected from the original dataset to represent the largest diversity possible in terms of human shape and to ensure that the meshes do not contain any irregularities (e.g., holes due to occlusions in the scanning process). To make the subjects assume the required T-pose, they were automatically rigged and posed using Mixamo (https://www.mixamo.com/). Body measurements of the subjects were computed using Trimesh, whereas the front and side rendered images were obtained using the script presented in Section “Methods”.

The same nomenclature introduced in the previous section holds also for this study to indicate the different configurations that were tested.

**Fig. 12:**
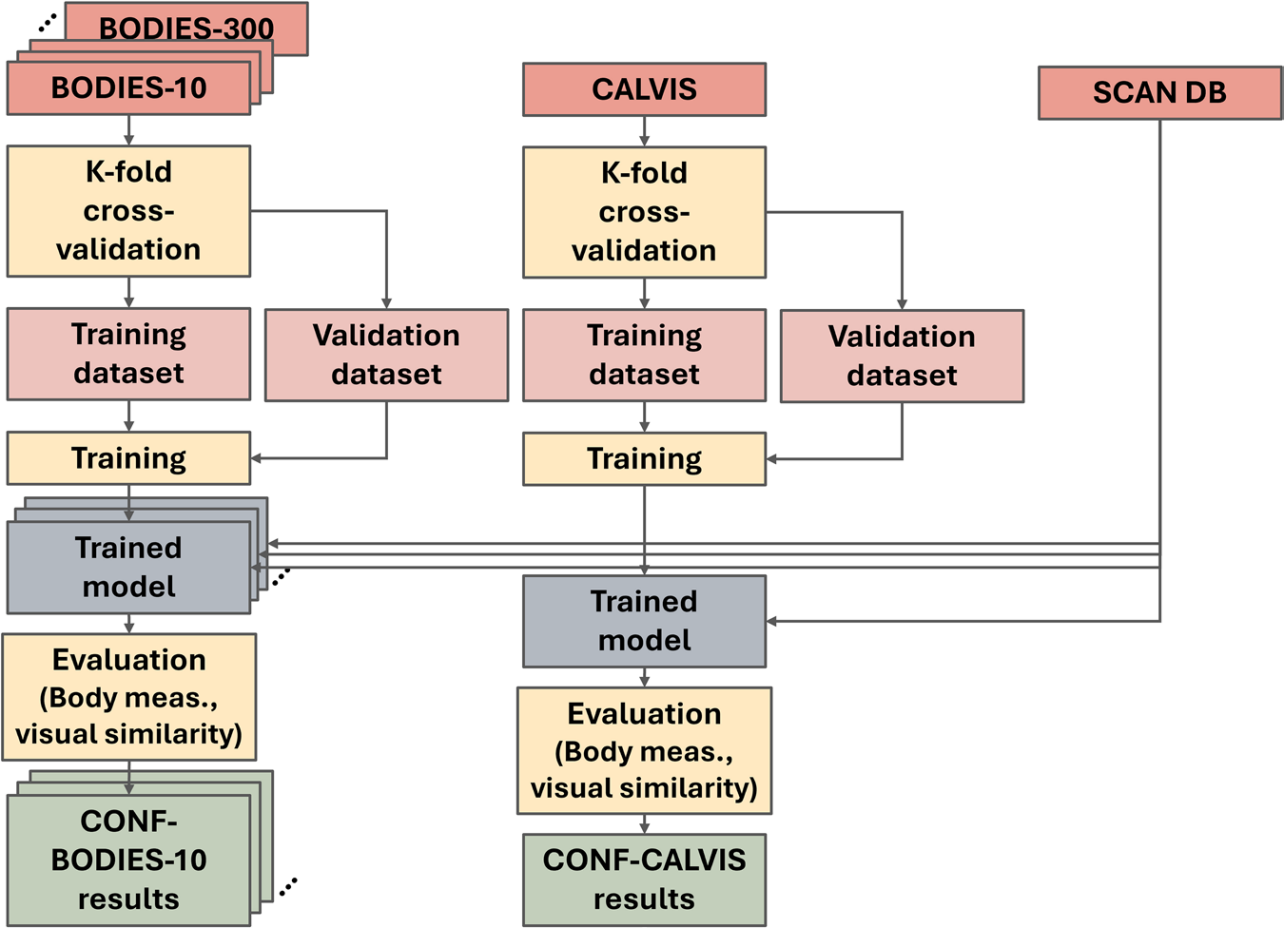
Schematic representation of the training and testing workflow adopted in Study 3.

The overview of how the datasets were employed for training and testing in this last study is depicted in Fig. 12.

### Metrics

In order to assess the performance of the framework in the three studies, it was chosen to evaluate the reconstruction error measured by comparing the generated 3D mesh with the corresponding ground truth. More specifically, the reconstruction error was computed by providing the framework with the front and side rendered images of 3D meshes of the subjects included in the test set and comparing the resulting 3D meshes (generated by inference using the framework) with the original ones. As suggested in^2^ and^11^, the reconstruction error was estimated in terms of distances between the 3D positions of the vertices, as well as of differences between the values of parameters and body measurements. More specifically the following metrics were computed:

- Per-parameter mean error (PME)^2^: this metric is computed as the MAE of the differences between the parameters in the meshes used as ground truth and the predicted ones.
- Per-vertex mean error^2^ (VME): this metric considers the MAE of the distances between the vertices in the meshes used as ground truth and the predicted ones.
- Body measurements^11^: this metric considers the errors between the ground truth and the reconstructed mesh for a given set of body measurements. Like in^11^, the following measurements were considered: *weight*, *height*, and circumferences of *chest*, *waist*, and *hip*.

As mentioned above, the three studies include cases in which the framework was trained and tested with different datasets. This means that the 3D meshes generated by the framework were represented using a parametric model and/or a number of parameters that differ from the meshes used as ground truth. In these cases, it was not possible to compare the two meshes using the VME and PME. In fact, computing VME would require comparing the positions of the vertices but this comparison was not allowed since the topologies (hence the number and position of the vertices) of the two meshes change between the SMPL and SUPR models. Moreover, it was not possible to identify a correspondence between the values of the parameters in the SMPL and SUPR models that could support the computation of the PME. Therefore to analyze the considered cases and support cross-comparisons, the reconstruction error was computed using the body measurements.

Finally, to complement Study 3, a qualitative metric was added to the analysis. More specifically, four subjects (two male and two female) were randomly picked from the Scan DB dataset, and 15 volunteers were asked to evaluate the visual similarity of the generated meshes with respect to the ground truth. A 1-to-5 Likert scale (ranging from *not at all* to *very similar*) was used.

The metrics were computed for the configurations described in Section “Experimental Studies” by splitting the samples contained in the considered datasets into two groups, i.e., male and female subjects, as proposed in^2^.

### Results

This section reports and analyzes the values of the metrics computed in the three studies.

#### Study 1 – Comparing SMPL and SUPR as training sets

Table 2 reports the values of PME and VME for the two configurations considered in this study (distinguishing between male and female subjects). It is possible to notice that the BODIES-10 dataset was able to improve the performance of the original framework, as both the PME and the VME were reduced when the framework was trained and tested with subjects represented with the SUPR model (i.e., the best results were obtained for CONF-2). However, it is worth outlining that these results were obtained by training the framework with different datasets, hence they are only partially comparable. Recently, a methodology has been proposed in^38^, which in principle could enable the conversion from SMPL to SUPR (and viceversa). However, the framework introduced in^38^ achieved the lowest performance when converting between SMPL and SUPR representations. Furthermore, that framework was trained on the AMASS and Motion-X datasets rather than on CALVIS, which may further affect conversion accuracy and introduce bias into the study results. Therefore, although future tools (or improvements of the current ones) might eventually enable reliable conversion between SMPL and SUPR representations, this remains, at present, a partially open issue, and the results obtained from the two models should still be considered only partially comparable. As the performance of such tools improves, it could become possible to convert CALVIS subjects from SMPL to SUPR, thus allowing the training set to include the same subjects expressed in both models. In this way, the results would become fully comparable.

**Table 2:** Study 1 – Reconstruction error in terms of PME and VME for the three configurations.

	

The highlighted cells indicate the minimum error among the configurations for each metric.

In order to supplement the above results, cross-comparisons were performed by testing the framework with the dataset not used for training (thus, for instance, CONF-1 was trained with CALVIS and tested with BODIES-10). Table 3 shows the results in terms of body measurements. It is possible to notice important differences between the behavior of the framework for the male and female subjects. For the male subjects, the performance of CONF-1 and CONF-2 is almost comparable, whereas, when analyzing the results for the female subjects, large differences can be observed. More specifically, the framework trained with the BODIES-10 dataset (CONF-2) and tested using the subjects in the CALVIS dataset (fourth row of Table 3) was able to generalize better than when trained with the CALVIS dataset (CONF-1) and tested with subjects in the BODIES-10 dataset (second row of Table 3), as it achieved more favourable results in terms of body measurements. This result may be related to the improved expressiveness of the SUPR model, which could allow the framework to learn better how to represent the bodies of females that are usually characterized by a higher complexity than the male ones.

**Table 3:** Study 1 – Reconstruction error in terms of body measurements for CONF-1 and CONF-2.

Configuration	Test	Height [*m*]	Weight [*kg*]	Chest [*m*]	Waist [*m*]	Hip [*m*]
CONF-1 (male)	BODIES-10	0.03083	2.47338	0.05537	0.04212	0.02351
CONF-1 (female)	BODIES-10	0.08490	14.5676	0.15170	0.15330	0.11080
CONF-2 (male)	CALVIS	0.03913	3.54828	0.20259	0.02516	0.02130
CONF-2 (female)	CALVIS	0.00780	0.82740	0.04910	0.02560	0.00850

#### Study 2 – Analyzing the Effect of Varying the Number of Parameters

Table 4 reports the results in terms of PME and VME for the framework trained and tested with subjects belonging to the same dataset (e.g., trained with the training set and tested with the test set of the BODIES-10 dataset). Results show that the reconstruction errors in terms of both PME and VME increase proportionally with the number of parameters.

**Table 4:** Study 2 – Reconstruction error in terms of PME and VME for the considered configurations.

	

Highlighted cells indicate the minimum error among the configurations for each metric.

**Fig. 13:**
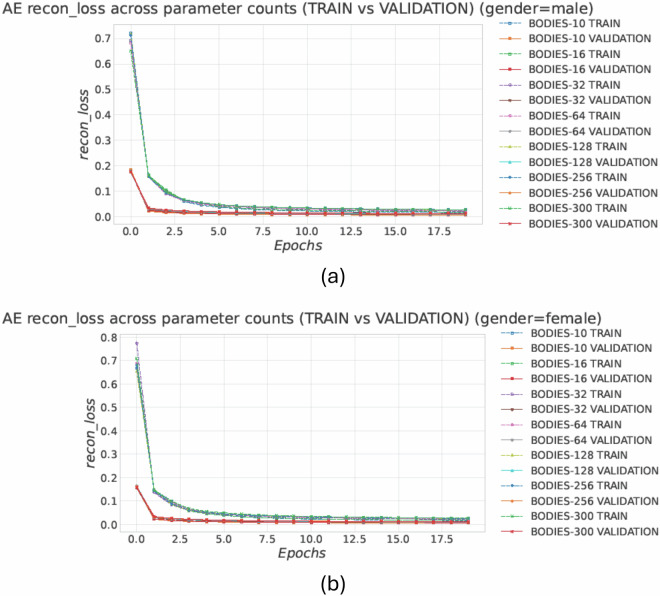
Auto-encoder reconstruction-loss learning curves across all parameter settings *N* ∈ {10, 16, 32, 64, 128, 256, 300}, reported separately for (**a**) male and (**b**) female subjects. Curves converge rapidly and train/validation trends remain consistent across *N*, indicating stable optimization at the feature-learning stage (minor train–validation differences can be attributed to stochastic regularization during training).

**Fig. 14:**
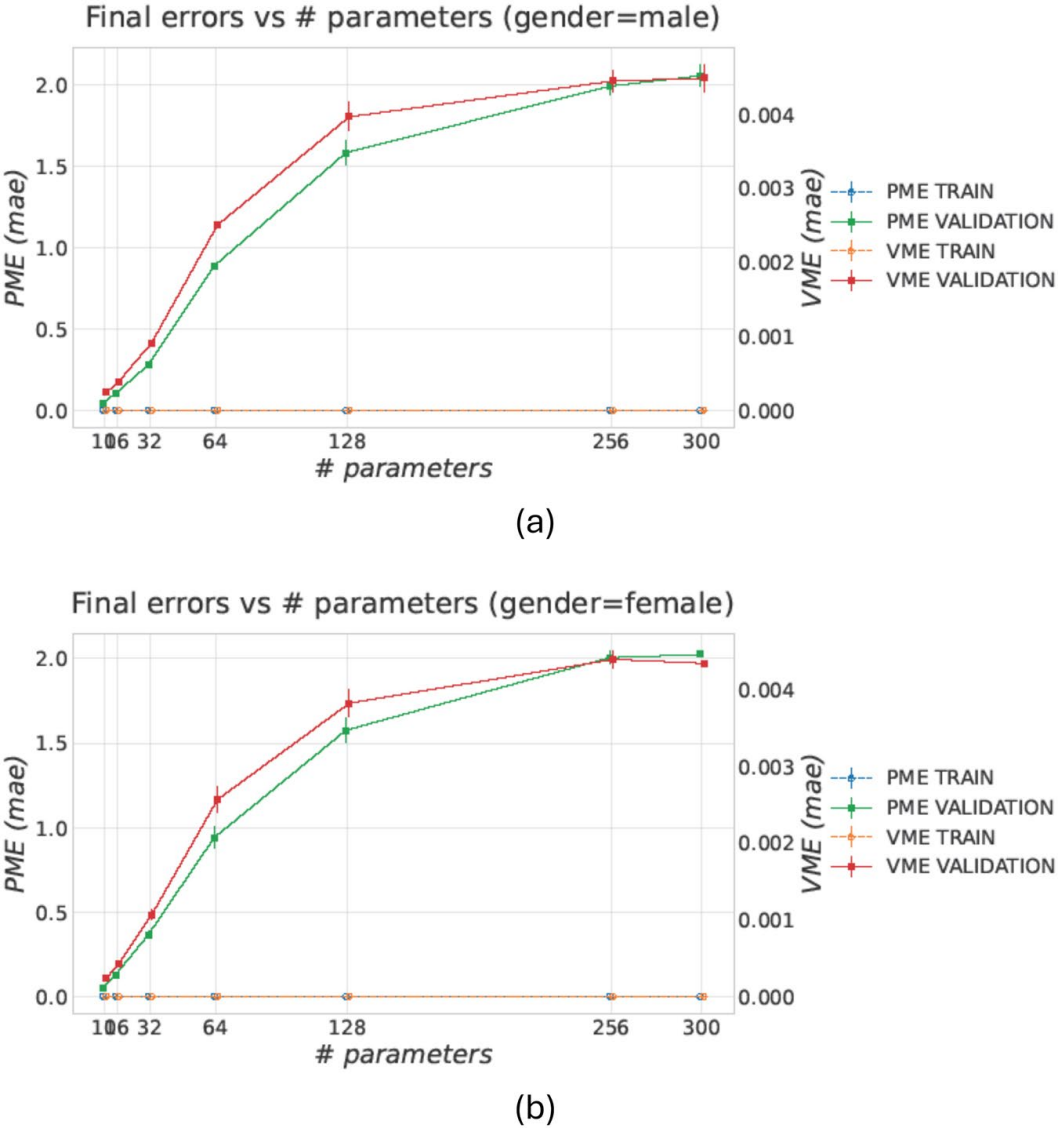
Final PME and VME (mean ± std across runs) as a function of the number of predicted shape parameters *N* reported separately for (**a**) male and (**b**) female subjects. Training errors are much smaller than validation errors on the reported scale, whereas validation errors increase markedly with *N*, revealing a growing generalization gap as the reconstruction task becomes higher-dimensional and less constrained by silhouette evidence.

As the number of predicted shape parameters *N* increases, reconstruction becomes more difficult: higher-order coefficients encode more localized deformations, which are only weakly constrained by two silhouettes and coarse anthropometric data (Table 4). With the fixed pipeline, the increased output dimensionality makes the regression phase more challenging and pushes the model into a higher variance regime. Figure 13 shows similar convergence between training and validation for the autoencoder across all *N*, suggesting that feature learning is not the main bottleneck; instead, Fig. 14 shows a growing gap between training and validation in PME/VME with *N*, indicating degraded generalization in the parameter prediction phase and a more difficult inference problem.

To complement the above analysis, the considered configurations were also evaluated through the body measurements. In this case, the framework trained, e.g., with BODIES-10, was used to reconstruct subjects belonging to the other dataset. Also in this case, errors increase for all the body measurements when the number of parameters is larger, regardless of which dataset is used for training. Conclusions similar to those already drawn regarding the complexity of the task also hold in this case. Detailed results for each configuration are given in the Appendix which is provided as supplemental material (available at https://tinyurl.com/2p9u3ufv).

**Fig. 15:**
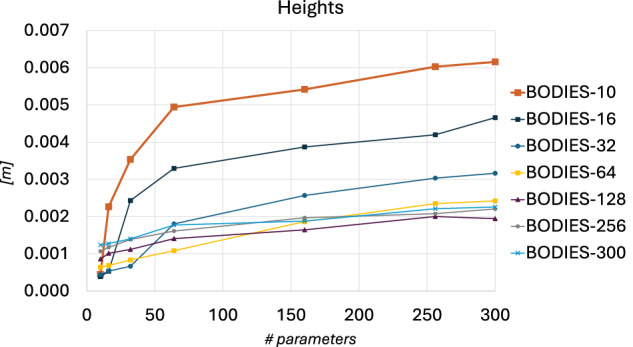
Plots showing the progression of the error in the considered body measurement (the height) computed for the female subjects as a function of the number of parameters used to describe them in the test set, for all the considered configurations. The Integral Value (IV) for a given configuration is the area under the corresponding plot.

To better analyze the behavior of the framework across several configurations, it was also computed the Integral Value (in the following referred to as IV) of the functions that describe the progression of a given metric depending on the number of parameters used to represent the subjects in the test set. For instance, Fig. 15 illustrates the progression of the error for a body measurement (i.e. the height) for all the considered configurations. The IV is the area under each plot.

The analysis of the IV could provide useful insights about the capability of the framework to generalize to other test sets. In fact, the lower this value is, the greater the generalization ability of the framework should be, since low values of IV result from smaller differences between the evaluated body measurements and the ground truth for all datasets used as test sets.

Analyzing the results in terms of IV for a given metric across the different configurations it can be observed that values decrease till a certain point, then they tend to increase again. For instance, the IV computed for the height of the male subjects is equal to 1.47716 for CONF-BODIES-10, then it reaches its minimum (i.e. 0.44544) for CONF-BODIES-128, and increases again reaching 0.54063 for CONF-BODIES-300. Similar trends are observed for the other metrics.

The decrease in the IV means that, overall, the framework trained with an increasing number of parameters performs better (i.e. generalize better) when tested on the other datasets. This outcome is in line with the previous observations. In fact, using a dataset with subjects described with a small number of parameters makes the framework specialize in a simple task, reducing its capacity to generalize for subjects that are described with a different number of parameters and present unseen details in the shape from those included in the training set. In contrast, a framework trained with a larger number of parameters has already learned how to represent fine details in the body shape and it can also predict better an increased number of parameters that allow the framework to model such details.

**Fig. 16:**
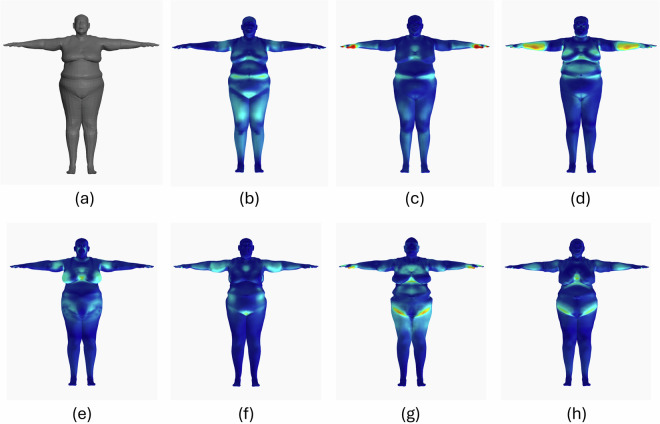
A subject of the BODIES-300 dataset as reconstructed by all the configurations considered in Study 2: (**a**) ground truth, (**b**) CONF-BODIES-10, (**c**) CONF-BODIES-16, (**d**) CONF-BODIES-32, (**e**) CONF-BODIES-64, (**f**) CONF-BODIES-128, (**g**) CONF-BODIES-256, and (**h**) CONF-BODIES-300.

However, as anticipated, the decrease in the IV is not consistent throughout all the configurations. In particular, for the BODIES-32/-64/-128 configurations, an inversion in this trend is generally observed. This behavior could be explained by the fact that the increase in the task complexity (due to the increase in the number of parameters) affects the performance of the framework, thus making the reconstruction error (and so the IV) increases as well. Fig. 16 shows how the configurations reconstructed the same subjects. More specifically, the subject serving as ground truth in this figure comes from BODIES-300, since it is the dataset that presents the greatest variability and number of details in the shape of the subjects by construction. To better visualize the spatial distribution of reconstruction errors across the generated 3D meshes, a heatmap-based visualization is employed in Fig. 16. More specifically, the magnitude of the distance between each vertex and the closest vertex in the ground-truth mesh is represented using a color gradient. In this way, high-error regions (in red) can be clearly distinguished from areas where the mesh closely matches the ground truth (in blue).

#### Study 3 – Evaluating the Training with SMPL and SUPR for Reconstructing Real Subjects

**Fig. 17:**
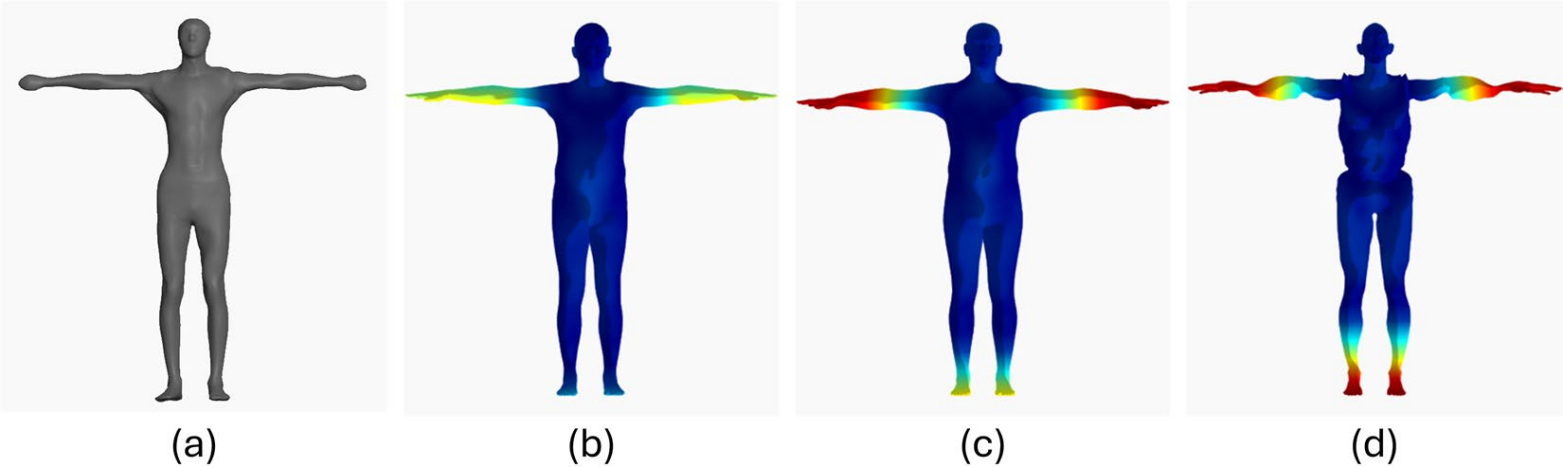
A subject of the Scan DB as reconstructed by different configurations considered in Study 3: (**a**) ground truth, (**b**) CONF-CALVIS, (**c**) CONF-BODIES-10, and (**d**) CONF-BODIES-16.

The results regarding body measurements for male and female subjects are reported in Tables 5 and 6, respectively. In this case, the error is generally characterized by an increasing trend for all the metrics. This result can be attributed to a domain-shift setting: the training data are synthetic (SUPR/SMPL-based), whereas the test set contains real scanned subjects from the Scan DB dataset, with different geometry statistics and mesh topology. In this regime, configurations predicting few shape parameters act as an implicit regularizer: the model has limited degrees of freedom and tends to produce conservative reconstructions closer to the template, which can be more robust out-of-distribution. Conversely, when trained to estimate more parameters, the framework gains higher flexibility and can enter a higher-variance regime, attempting to imitate scan-specific details that are not reliably supported by silhouettes and training priors, thus introducing artifacts in the reconstructed mesh. This behavior is illustrated in Fig. 17, which compares the same subject reconstructed by frameworks trained with the CALVIS, BODIES-10, and BODIES-16 datasets. This interpretation is also consistent with the generalization degradation observed in Study 2 (Fig. 14), which is expected to be amplified under domain shift.

**Table 5:** Study 3 – Reconstruction error in terms of body measurements for configurations with male subjects.

	

Highlighted cells indicate the minimum error among the configurations for each metric. Values reported using a bold font represent measurements in which configurations trained with the BODIES dataset outperformed CALVIS.

**Table 6:** Study 3 – Reconstruction error in terms of body measurements for configurations with female subjects.

	

Highlighted cells indicate the minimum error among the configurations for each metric. Values reported using a bold font represent measurements in which configurations trained with the BODIES dataset outperformed CALVIS.

Unfortunately, the amount of artifacts introduced in the mesh, especially for female subjects, does not make it possible to compute the reconstruction errors for all the subjects in the test set, since the script fails to automatically identify the points in the mesh to be used for performing the measurements. For this reason, Tables 5 and 6 report only the errors for those configurations in which it was possible to compute the metrics for at least 80% of the subjects.

Comparing CONF-BODIES-10/16 with CONF-CALVIS (measurements highlighted with a bold font in Tables 5 and 6 represent cases in which the comparison between the two datasets, i.e., BODIES and CALVIS, was in favor of the former), it is possible to notice that, for the male subjects, the results are almost comparable, whereas, for the female subjects, training the framework with the BODIES dataset allowed it to perform better. This is an interesting result, since, as already observed in the previous sections, the female subjects are generally characterized by a higher complexity.

**Fig. 18:**
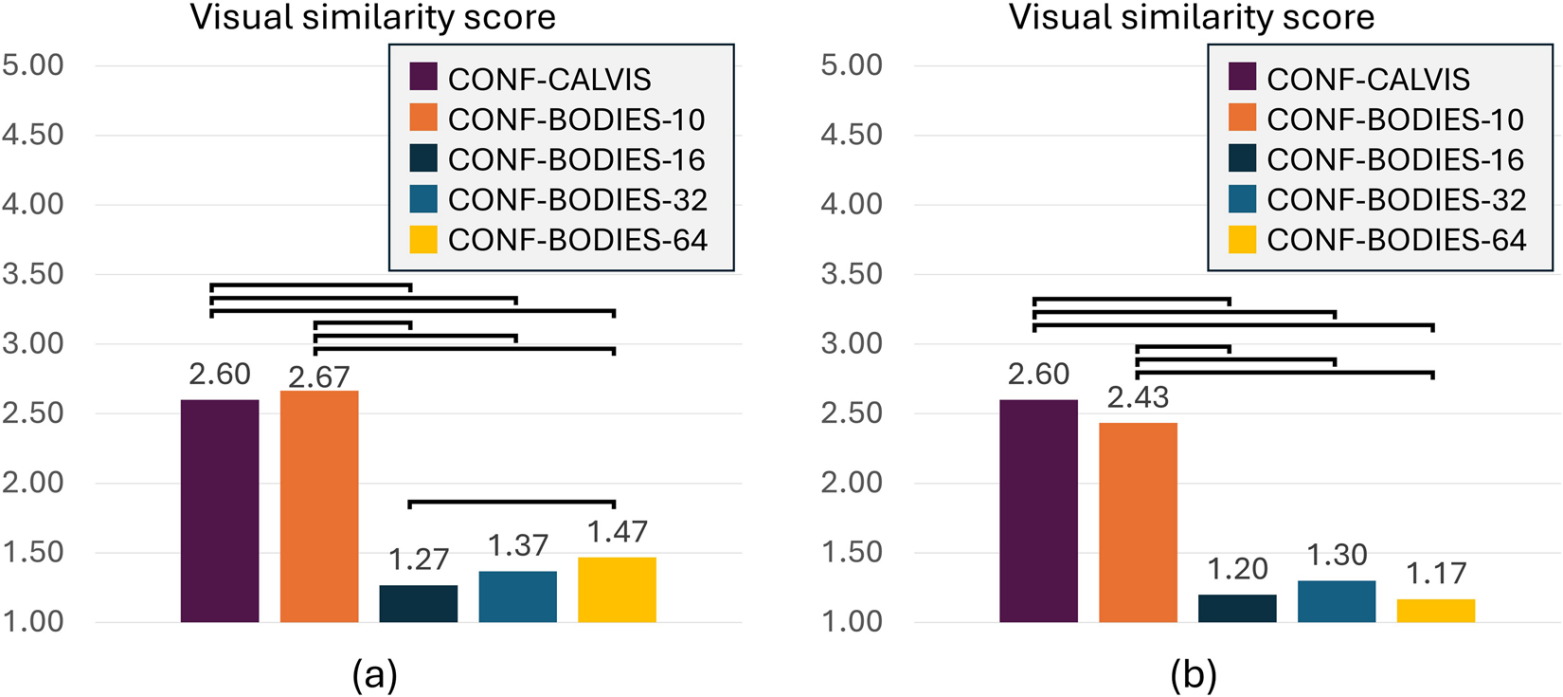
Study 3 – Visual similarity scores for configurations with (**a**) male and (**b**) female subjects. Statistically significant pairwise comparisons are denoted with brackets connecting the corresponding pairs.

Regarding visual similarity, Fig. 18 reports the similarity scores assigned by the participants in the user study. Statistical significance was assessed using the Friedman test and the Wilcoxon signed-rank test for paired comparisons.

Consistent with the results presented above, the analysis was focused only on the configurations capable of generating subjects with a limited number of artifacts. Therefore, participants were asked to evaluate subjects generated with CONF-CALVIS and CONF-BODIES-10 to CONF-BODIES-64. It can be observed that, in general, participants rated the visual similarity as lower than 3 for all the configurations, confirming the limitations of the considered framework in reconstructing subjects in the Scan DB dataset. Comparing these results with the objective measurements reported in Tables 5 and 6, it is worth noting that CONF-CALVIS and CONF-BODIES-10 are the configurations that received the highest similarity ratings, even though they do not achieve the best performance in terms of body measurements. This result indicates that, from an objective viewpoint, the generated meshes may be reasonably accurate with respect to the considered set of body measurements. However, the number of artifacts introduced during reconstruction from configurations characterized by a higher number of parameters distorted the meshes in ways that led participants to perceive them as less similar overall.

### Use Cases

The results discussed in the previous sections demonstrate the improvements, in terms of reconstruction error, introduced by the use of the SUPR parametric model for representing complex human bodies. Such improvements are crucial in several scenarios, particularly when high-fidelity 3D avatars are required to maximize the effectiveness of the applications.

As a matter of example, in the fashion industry, highly detailed 3D avatars describing the body of customers can enable realistic virtual try-on experiences and accurate garment simulations^16^. In the healthcare domain, realistic 3D human models are essential for improving user experience and expanding functionality in applications such as surgical simulation, patient-specific modeling, and training of procedures that demand precise anatomical accuracy^39^.

Methods such as^26^ and^40^ are able to generate photorealistic and animatable 3D humans from a single photo. These works do not focus solely on visual appearance, but also aim to reconstruct body geometry consistent with the proportions in the input image. In the context of entertainment, this can lead, e.g., to avatars that truly resemble the player’s character rather than merely looking good.

Finally, in the virtual heritage domain, studies such as^41^ have demonstrated that the use of high-fidelity avatars significantly enhances users’ sense of presence and immersion in virtual reality applications, where avatars are leveraged to guide users in the exploration and interaction with virtual artifacts.

## Data Availability

The BODIES dataset is available for download at^28^.

## Code availability

The source code that can be used to run the experiments described in this paper is available at https://tinyurl.com/kmrk4rwj.
